# Complete mitochondrial genome of *Minla ignotincta* (Passeriformes: Timaliidae)

**DOI:** 10.1080/23802359.2016.1144099

**Published:** 2016-02-10

**Authors:** Bo Li, Yongfang Yao, Diyan Li, Qingyong Ni, Mingwang Zhang, Meng Xie, Huailiang Xu

**Affiliations:** a College of Life Science, Sichuan Agricultural University, Ya’an, China;; b College of Animal Science and Technology, Sichuan Agricultural University, Chengdu, China

**Keywords:** *Minla ignotincta*, mitochondrial genome, phylogenetic analysis

## Abstract

We sequenced the complete mitochondrial genome from *Minla ignotincta*. The genome sequence was 17 868 bp in length, and the gene arrangement and contents were identical to those of previously reported Timaliidae mitochondrial genomes. The overall base composition of the mitogenome is biased toward A + T content at 54.04%. All protein-coding genes (PCGs) began with ATG. Nine of the 13 PCGs used complete (TAA) or incomplete (TA or T) stop codon, while *ND1* and *ND5* ended with AGA, *COI* ended with AGG and *ND6* with TAG. All the genes in *M. ignotincta* were distributed on the H-strand, except for the ND6 subunit gene and eight tRNA genes which were encoded on the L-strand.

The Timaliidae, generally known as the babblers, was a diverse family of oscine passerine birds that traditionally includes about 275 species in 50 genera (Dickinson & Christidis 2003). Most molecular phylogenetic work have focused on subsets of the Timaliidae (Pasquet et al. 2006; Zhang et al. 2007; Yeung et al. 2011). Till now, there was little information about the complete mitochondrial genomes of *M. ignotincta* in GenBank. In our study, the complete mitogenome of *M.ignotincta* was determined, which might provide some molecular data for the research on the relationships of some babblers.

*Minla ignotincta* (specimen voucher B43) was obtained from Ya’an, Sichuan province of China (N29.98°, E103.01°) and identified by its morphological characteristics. The complete mitochondrial genome sequence of *M. ignotincta* was amplified and sequenced by 18 pairs of primers with normal LA-PCR and PCR methods. The mitogenome of *M. ignotincta* has been deposited in Genbank (accession no. KT995474). We constructed the phylogeny by Bayesian inference (BI) using Mrbayes 3.1.1 (Huelsenbeck & Ronquist 2001).

Similar to other Timaliidae mitogenomes, the complete mtDNA sequence of *M. ignotincta* (17 868 bp in length) had 13 typical protein-coding genes (PCGs), 22 tRNA genes, two rRNA genes (12S rRNA and 16SrRNA) and two control region (*D-loop1* and *D-loop2*) (Zhang et al. 2014; Zhou et al. 2015). The base composition of mtDNA is 30.05% A, 23.99% T, 31.76% C and 14.20% G, so the percentage of A + T (54.04%) was slightly higher than G + C. In 13 PCGs, the shortest one was *ATP8* gene (168 bp) and the longest one was the *ND5* gene (1818 bp). All PCGs began with ATG. Of the 13 PCGs, nine of PCGs used complete (TAA) or incomplete (TA or T) stop codon, while *ND1* and *ND5* ended with AGA, *COI* ended with AGG and *ND6* with TAG. The *12S rRNA* (982 bp) and *16S rRNA* (1600 bp) genes were located between the *tRNA^phe^* and *tRNA^Leu(UUR)^* genes, which was separated by the *tRNA^Val^* gene. The inferred secondary structures of 21 tRNAs (excluding *tRNA^Ser(AGY)^*) of *M. ignotincta* were all conformed to the common structural features of mitochondrial tRNAs. *D-loop1* and *D-loop2* were located in two different locations of mitogenome. There was eight tRNA genes (*tRNA^Gln^*, *tRNA^Ala^*, *tRNA^Asn^*, *tRNA^Cys^, tRNA^Tyr^*, *tRNA^Ser(UCN)^*, *tRNA^Pro^* and *tRNA^Glu^*) and one PCG (*ND6*) encoded on the L-strand, other PCGs encoded on the H-strand.

As shown in Figure 1, 10 Timaliidae species clustered into two groups. A separate *M. ignotincta* was the sister lineage to the clade formed by the species of genus *Leiothrix* with more than 96% of posterior probability, which was consistent with BI analysis results of Gelang et al. (2009) and Moyle et al. (2012). Therefore, we hold that the genomic data of *M. ignotincta *was trustworthy.

**Figure 1.**
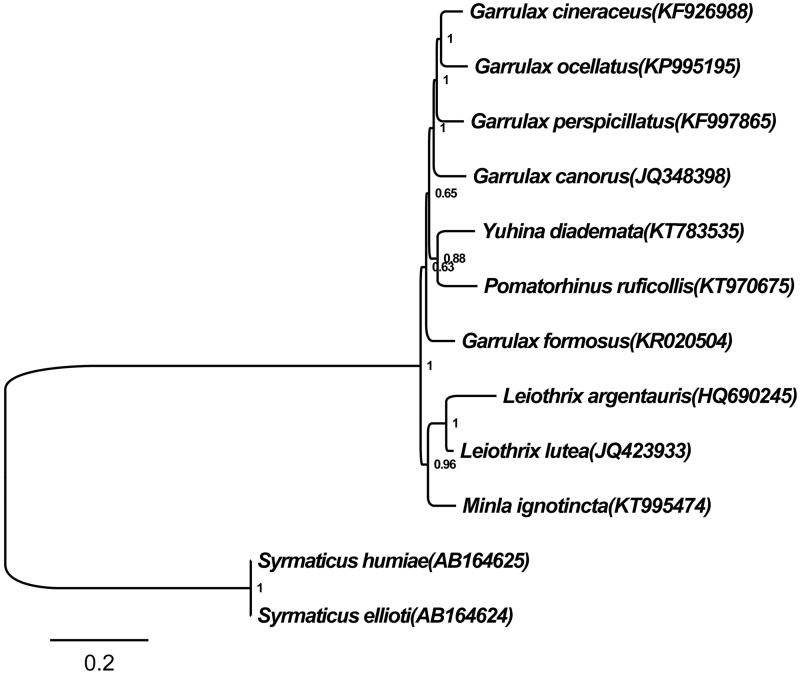
Bayesian tree based on combining 13 protein-coding gene sequences of ten Timaliidae and two *Syrmaticus* birds. The Bayesian tree was reconstructed with general-time-reversible (GTR) model, and the Markov chains for 1 000 000 generations. Numbers at node of the tree branches represent posterior probability. Two *Syrmaticus* birds were used as the outgroup.

## References

[CIT0001] Dickinson EC, Christidis L. 2003 The Howard & Moore complete checklist of the birds of the world, vol. 2: Passerines. 3rd ed. Eastbourne: Aves Press.

[CIT0002] Huelsenbeck JP, Ronquist F. 2001 MRBAYES: Bayesian inference of phylogenetic trees. Bioinformatics. 17:754–755. 11524383 10.1093/bioinformatics/17.8.754

[CIT0003] Gelang M, Cibois A, Pasquet E, Olsson U, Alström P, Ericson PGP. 2009 Phylogeny of babblers (Aves, Passeriformes): major lineages, family limits and classification. Zool Scr. 38:225–236.

[CIT0004] Moyle RG, Andersen MJ, Oliveros CH, Steinheimer FD, Reddy S. 2012 Phylogeny and biogeography of the core babblers (aves: timaliidae). Syst Biol. 61:631–651. 22328569 10.1093/sysbio/sys027

[CIT0005] Pasquet E, Bourdon E, Kalyakin MV, Cibois A. 2006 The fulvettas (Alcippe, Timaliidae, Aves): a polyphyletic group. Zool Scr. 35:559–566.

[CIT0006] Yeung CKL, Lin RC, Lei F, Robson C, Hung LM, Liang W, Zhou F, Han L, Li SH, Yang X. 2011 Beyond a morphological paradox: complicated phylogenetic relationships of the parrotbills (Paradoxornithidae, Aves). Mol Phylogenet Evol. 61:192–202. 21704175 10.1016/j.ympev.2011.06.004

[CIT0007] Zhang H, Li Y, Wu X, Xue H, Yan P, Wu X. 2016 The complete mitochondrial genome of garrulax perspicillatus (passeriformes, timaliidae). Mitochondrial DNA. 27:1265–1266. 25090386 10.3109/19401736.2014.945548

[CIT0008] Zhang S, Yang L, Yang X, Yang J. 2007 Molecular phylogeny of the yuhinas (Sylviidae: Yuhina): a paraphyletic group of babblers including Zosterops and Philippine Stachyris. J Ornithol. 148:417–426.

[CIT0009] Zhou YY, Qi Y, Yao YF, Huan ZJ, Li DY, Xie M, Ni QY, Zhang MW, Xu HL. 2015 Characteristic of complete mitochondrial genome and phylogenetic relationship of garrulax sannio (passeriformes, timaliidae). Mitochondrial DNA. 26:1–2. 26099976 10.3109/19401736.2015.1060454

